# Lightweight U-Net for Blood Vessels Segmentation in X-Ray Coronary Angiography

**DOI:** 10.3390/jimaging11040106

**Published:** 2025-03-30

**Authors:** Jesus Salvador Ramos-Cortez, Dora E. Alvarado-Carrillo, Emmanuel Ovalle-Magallanes, Juan Gabriel Avina-Cervantes

**Affiliations:** 1 Telematics and Digital Signal Processing Research Groups (CAs), Engineering Division, Campus Irapuato-Salamanca, University of Guanajuato, Carretera Salamanca-Valle de Santiago km 3.5 + 1.8 km Comunidad de Palo Blanco, Salamanca 36885, Mexico; js.ramoscortez@ugto.mx; 2 Faculty of Engineering, Universidad Virtual del Estado de Guanajuato, Hermenegildo Bustos 129 A Sur Centro, Purísima del Rincón 36400, Mexico; doalvarado@uveg.edu.mx; 3 Dirección de Investigación y Doctorado, Facultad de Ingeniería y Tecnología, Universidad La Salle Bajío, Av. Universidad 602. Col. Lomas del Campestre, León 37150, Mexico

**Keywords:** blood vessel, U-Net, lightweight, segmentation, X-ray coronary angiography

## Abstract

Blood vessel segmentation in X-ray coronary angiography (XCA) plays a crucial role in diagnosing cardiovascular diseases, enabling a precise assessment of arterial structures. However, segmentation is challenging due to a low signal-to-noise ratio, interfering background structures, and vessel bifurcations, which hinder the accuracy of deep learning models. Additionally, deep learning models for this task often require high computational resources, limiting their practical application in real-time clinical settings. This study proposes a lightweight variant of the U-Net architecture using a structured kernel pruning strategy inspired by the Lottery Ticket Hypothesis. The pruning method systematically removes entire convolutional filters from each layer based on a global reduction factor, generating compact subnetworks that retain key representational capacity. This results in a significantly smaller model without compromising the segmentation performance. This approach is evaluated on two benchmark datasets, demonstrating consistent improvements in segmentation accuracy compared to the vanilla U-Net. Additionally, model complexity is significantly reduced from 31 M to 1.9 M parameters, improving efficiency while maintaining high segmentation quality.

## 1. Introduction

Accurate segmentation of the coronary vessels in X-ray coronary angiography (XCA) images is crucial to the diagnosis of cardiovascular disease, particularly coronary artery disease (CAD). It enables clinicians to accurately identify and measure the degree of stenosis (narrowing) in the arteries, assess circulatory disturbances, and plan appropriate interventions, such as stent placement or bypass surgery. Precise vessel depictions aid in identifying coronary stenosis and facilitate more informed diagnostic and therapeutic decisions [1].

However, several challenges make coronary vessel segmentation difficult [2], due to the following factors:

1. Low signal-to-noise ratio: XCA images often have a poor signal-to-noise ratio, making distinguishing vessel edges from the background difficult.
2. Interfering background structures: Other anatomical structures and medical devices can obscure the vessels and complicate segmentation.
3. Non-uniform illumination: Uneven lighting in the images can lead to inconsistent vessel appearances.
4. Artifacts and noise: Various artifacts and noise can further degrade image quality and hinder segmentation.
5. Vessel junctions and bifurcations: Segmenting vessels at branching points is particularly difficult.

Deep learning-based models, particularly U-Net [3], have been widely adopted for medical image segmentation due to their ability to automatically learn hierarchical features from input data, ranging from low-level features such as edges to high-level features like shapes and patterns. For this reason, several variants have been proposed to customize the model for the specific task, such as integrating advanced layers that aim to extract particular features by adding additional weights and layers, compromising the number of parameters and, therefore, the training time.

Despite the growing interest in real-time processing and deployment in resource-constrained environments such as portable diagnostic devices, there is no systematic evaluation of lightweight U-Net architectures for XCA vessel segmentation. Specifically, no prior work has investigated kernel pruning strategies to reduce computational complexity while maintaining segmentation accuracy. This presents a critical research gap in the optimization of deep learning models for XCA analysis.

This study presents an evaluation of lightweight U-Net architectures for coronary vessel segmentation in XCA images. By reducing the number of filters in the U-Net model, the aim is to balance computational efficiency and segmentation accuracy. To the best of the author’s knowledge, this is the first study to apply a kernel pruning strategy to U-Net for XCA vessel segmentation. The main contributions are as follows:

1. A systematic evaluation of lightweight U-Net models for coronary vessel segmentation.
2. An analysis of the trade-off between model complexity and segmentation accuracy.
3. Demonstration of the feasibility of kernel pruning for optimizing U-Net performance in XCA image segmentation.

The remainder of this paper is organized as follows: Section 2 reviews the related work on XCA image segmentation. Section 3 describes the proposed pruning methodology, the modified U-Net architecture, and details the datasets and evaluation metrics. Section 4 presents the experimental setup, discusses the results, and compares the performance with baseline models. Finally, Section 5 summarizes the findings and outlines directions for future research.

## 2. Related Work

The primary objective of medical image segmentation is to delineate various regions or structures within an image precisely. This process involves obtaining continuous contour and edge information for specific areas via pixel-level classification. Deep learning models, particularly convolutional neural networks (CNNs), can automatically learn hierarchical features from input data, ranging from low-level features like edges to high-level features like shapes and patterns. This is crucial for detecting complex structures in medical images.

Due to its simplicity and adaptability, the U-Net framework was selected as the foundational structure among various deep learning models for medical image segmentation. It utilizes an encoder–decoder structure, a widely used and favored model in deep learning. In this model, the encoder extracts essential image features at different scales, and the decoder restores the original image dimensions to produce the final segmentation results.

For this reason, numerous beneficial structures have been proposed to enhance their efficacy, including normalization [4], residual blocks [5], dense blocks [6], inception blocks [7], dilated convolution [8], attention modules [9], and more.

Gonçalves et al. [10]. presented a survey on attention mechanisms for medical applications, in which they studied how different attention mechanisms enable the modeling of comprehensive contextual information across various medical image modalities, including cardiac image segmentation. The computational complexity of these approaches typically grows quadratically with respect to the spatial size of the feature maps. Therefore, significant computational load is introduced during both the training and inference stages.

Considering this, at the expense of spatial resolution but with increased computational cost (i.e., number of parameters), Yeung et al. [11] proposed a focus attention gate for polyp segmentation. This gate uses the deepest feature maps of the U-Net to refine the incoming signals from the encoding network via long-range skip connections, highlighting specific features and regions of the image that are integrated into the decoding network.

Particularly for coronary artery segmentation, Iyer et al. [12] introduced the angiographic processing network (APN) as a preprocessing learnable step that improves segmentation performance. Thus, an end-to-end pipeline encompassing image preprocessing and segmentation for angiographic images of coronary arteries was tested using the U-Net as a segmentation model. The APN comprised several 3×3 and 5×5 convolutional layers, forming a six-layer model. Yang et al. [13] developed a CNN based on U-Net to segment the major branches of the coronary arteries. They focus their research on replacing the encoder module of the U-Net with one of the most popular networks for image classification, such as ResNet101 [5], DenseNet121 [6], or InceptionResNet-v2 [14]. Similarly, Mahjoub et al. [15] compared the performance of the Vanilla U-Net against employing a ResNet101 and DensNet121 as an encoder. Following this line of thought, Li and Fan [16] included as an encoder the lightweight backbone GhostNetV2 [17] that has 16, 24, 40, 80, and 160 filters in each layer, respectively. On the other hand, Zhao et al. [18] integrated a feature pyramid with a U-Net++ model [19] to automatically segment coronary arteries. This approach leaves the encoder intact and modifies the decoder stage by adding the feature pyramid layers to extract additional features during the upsampling.

These models often add more layers or modify the Vanilla U-Net architecture to improve performance. Moreover, even though U-Net is a large model that would benefit from compression, it is hardly considered in pruning studies [20,21]. Therefore, this paper explores removing filters before training from the Vanilla U-Net to train lightweight models that start with 16 and 32 instead of 64. This strategy reduces from 31 M to 1.9 M parameters, up to 16× smaller models without compromising the segmentation results.

## 3. Method

### 3.1. U-Net Model

The U-Net architecture, proposed by Ronneberger et al. [3], is the standard model for image segmentation tasks. The base model (Vanilla U-Net) incorporates an *encoder* (*E*) that consists of a contraction path that extracts hierarchical features from the input image X and a *decoder* which is an expansion path that reconstructs the output Y by upsampling and combining features from *E* (using skip connections).

The U-Net model can be understood as a mathematical function that maps an input image X to an output image Y. This can be expressed as:

(1) Y=D(E(X)).

On the one hand, the *encoder E* consists of a convolutional architecture comprising two consecutive convolutions, with a filter of size 3×3. Each one is followed by a *rectified linear unit* (ReLU) activation function and a *max pooling* operation of size 2×2 with a *stride* of two for descent. For each down-sampling block, the number of filters is doubled. Four down-sampling blocks exist, with 64, 128, 256, and 512 filters, respectively.

The *encoding* process can be expressed as:

(2) $$E\left(X\right)=\left\{F_{1},F_{2},F_{3},F_{4}\right\},$$

where $F_{i}$ are the feature maps at level *i*, obtained by:

(3) $$F_{i}=\left\{\begin{array}{cc} f_{\mathrm{pool}}\left(f_{\mathrm{relu}}\left(f_{\mathrm{conv}}\left(X\right)\right)\right) & \mathrm{if}\;i=1,\\ f_{\mathrm{pool}}\left(f_{\mathrm{relu}}\left(f_{\mathrm{conv}}\left(F_{i-1}\right)\right)\right) & \mathrm{if}\;i=2,3,4. \end{array}\right.$$

where $f_{\mathrm{conv}}\left(\cdot \right)$ is the convolution operation, $f_{\mathrm{relu}}\left(\cdot \right)$ is the ReLU activation function, which introduces nonlinearity, and $f_{\mathrm{pool}}\left(\cdot \right)$ is the max-pooling downsampling operation.

On the other hand, the *decoder* or expansion path upsamples the feature map obtained in the *encoder* part. Subsequently, 2×2 convolutions are applied iteratively, halving the number of filters. At each level, they are connected by concatenation with the *encoder* feature map, followed by two consecutive convolutions, with a filter of size 3×3 and the ReLU activation function. In the final part, a 1×1 convolution is implemented to preserve resolution but reduces the filters to *n*, which are the classes to predict.

The *decoding* process can be expressed as:

(4) $$D\left(E\right)=\left\{{\hat{F}}_{4},{\hat{F}}_{3},{\hat{F}}_{2},{\hat{F}}_{1}\right\},$$

where ${\hat{F}}_{i}$ are the feature maps reconstructed at level *i*, obtained by:

(5) $${\hat{F}}_{i}=\left\{\begin{array}{cc} f_{\mathrm{conv}}\left(f_{\mathrm{relu}}\left(f_{\mathrm{concat}}\left(f_{\mathrm{up}}\left(E_{4}\right),F_{4}\right)\right)\right) & \mathrm{if}\;i=4,\\ f_{\mathrm{conv}}\left(f_{\mathrm{relu}}\left(f_{\mathrm{concat}}\left(f_{\mathrm{up}}\left({\hat{F}}_{i+1}\right),F_{i+1}\right)\right)\right) & \mathrm{if}\;i=3,2,1, \end{array}\right.$$

where $f_{\mathrm{up}}\left(\cdot \right)$ is the *up-convolution* (or transposed convolution) used to increase spatial resolution, $f_{\mathrm{concat}}\left(\cdot ,\cdot \right)$ denotes the concatenation of the *encoder* and *decoder* feature maps at the corresponding level, $f_{\mathrm{relu}}\left(\cdot \right)$ is the ReLU activation function and $f_{\mathrm{conv}}\left(\cdot \right)$ is the convolution operation, which refines the reconstructed feature maps.

### 3.2. Pruning Process

Based on the Lottery Ticket Hypothesis [22], which states that a randomly initialized neural network contains a subnetwork that, when trained in isolation, can match the test accuracy of the original network, a filter pruning technique was used to compress the vanilla U-Net by removing entire filters from each convolutional layer by a factor α={2,4}. This approach offers advantages over creating sparse connections or other convolution techniques due to its straightforward implementation. Moreover, filter pruning reduces the number of filters, leading to a more compact model that can be efficiently implemented on mobile hardware.

**Figure 1:**
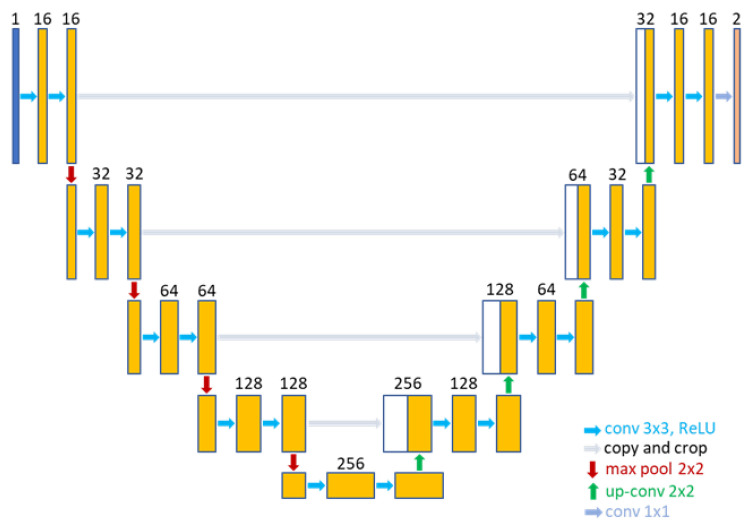
U-Net filter reduction for U-Net-16. This subnetwork contains 16, 32, 64, and 128 filters, respectively, and a latent space of 256 filters.

In this manner, the feature maps of each encoding block are reduced by this factor, leading to a subnetwork with 16, 32, 64, and 128 filters for α=4. Thus, the model parameters are reduced by 16× (1.9 M parameters), and a subnetwork with 32, 64, 128, and 256 filters for α=2, with a parameter reduction of 4× (7.7 M parameters). Hereafter, U-Net-16 and U-Net-32, respectively. Figure 1 illustrates the filter-removing process from the U-Net model.

### 3.3. Datasets

This research employed two datasets. The X-ray coronary angiography database (DCA1) [23] from the Cardiology Department of the Mexican Social Security Institute and the database ICA [18] were used in this study. The first dataset consists of 134 X-ray coronary angiograms and their corresponding ground-truth images, outlined by an expert cardiologist. Each angiogram is a 300×300-pixel grayscale image in PGM (portable gray map) format. The second database consists of a study involving 99 patients. The images were scanned at a size of 512×512 pixels, with a variable pixel spacing of 0.258 to 0.390 mm. The dataset consists of 616 coronary angiography images and their respective 616 segmentation masks, or *ground-truth* images. Of the 616 coronary angiography images, 405 depict the left coronary artery (LCA), and 211 represent the right coronary artery (RCA), with the same number of segmentation masks.

**Figure 2:**
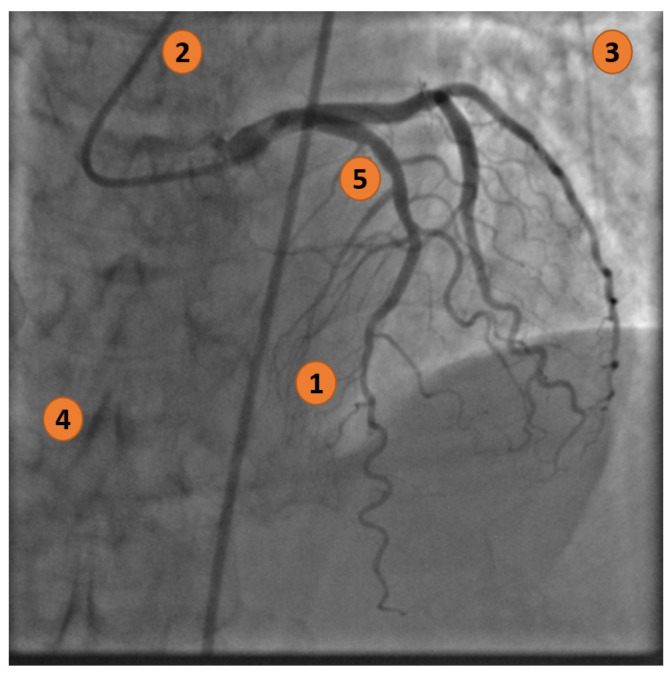
X-ray coronary artery with the following characteristics: (1) Zones with a low signal-to-noise ratio; (2) Interfering background structures (catheter); (3) Non-uniform illumination; (4) Artifacts and noise; and (5) Vessel junctions and bifurcations.

Figure 2 highlights five main visual complications when examining an XCA image (from the ICA dataset).

### 3.4. Evaluation Metrics

Six metrics were employed to compare the models’ performance in the vessel segmentation task in XCA images: intersection over union (IoU), dice score (DICE), accuracy (ACC), precision (PREC), recall (REC), and F1-score (F1). These metrics are commonly used in medical image segmentation tasks to evaluate spatial overlap (IoU, Dice) and classification performance (precision, recall, F1), especially in class-imbalanced settings such as vascular segmentation [3,24,25,26,27].

The IoU measures the overlap between the predicted segmentation mask and the ground truth mask. It is defined as:

(6) $$IoU=\frac{TP}{TP+FP+FN}.$$

where TP (true positives) represent correctly segmented vessel pixels, FP (false positives) corresponds to non-vessel pixels incorrectly classified as vessels, and FN (false negatives) are vessel pixels incorrectly classified as non-vessel.

The dice score, which quantifies the similarity between two sets:

(7) $$DICE=\frac{2TP}{2TP+FP+FN}.$$

This metric is particularly sensitive to small segmented structures and provides a balance between precision and recall.

Accuracy measures the overall correctness of the segmentation by considering both vessel and non-vessel pixels:

(8) $$ACC=\frac{TP+TN}{TP+TN+FP+FN},$$

where TN (true negatives) denotes correctly classified non-vessel pixels.

Precision, also known as positive predictive value (PPV), evaluates the proportion of correctly predicted vessel pixels relative to all predicted vessel pixels:

(9) $$PREC=\frac{TP}{TP+FP}.$$

A high precision value indicates a low rate of false positive classifications.

Recall, also referred to as sensitivity or true positive rate (TPR), measures the model’s ability to correctly identify vessel pixels:

(10) $$REC=\frac{TP}{TP+FN}.$$

A high recall value suggests that most vessel pixels are successfully segmented.

The F1-score is the harmonic mean of precision and recall, providing a balanced measure between false positives and false negatives, and is particularly useful when there is an imbalance between the vessel and background pixels. This metric is defined as:

(11) $$F1=\frac{2\cdot PREC\cdot REC}{PREC+REC}$$

### 3.5. XCA Blood Vessel Segmentation

**Figure 3:**
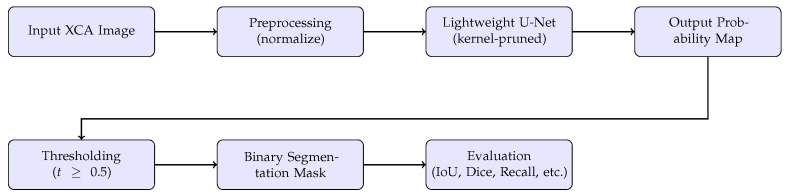
Segmentation pipeline using the Vanilla U-Net and the lightweight U-Nets.

The XCA blood vessel segmentation process using the proposed lightweight U-Net consists of four main stages, as illustrated in Figure 3:

1. Preprocessing: Each XCA image pixel normalized to a [0,1] range to ensure consistency in training. No additional filtering or vessel enhancement techniques are applied.
2. Training and prediction: The input to the model consists of the normalized XCA image and its corresponding binary ground truth mask. During training, the model minimizes a pixel-wise loss function (binary cross-entropy), learning to predict the probability that each pixel belongs to a blood vessel. During inference, the model outputs a probability map, where each pixel value indicates the likelihood of vessel presence.
3. Postprocessing: A fixed threshold (t=0.5) is applied to the output probability map to produce a binary segmentation mask. No additional morphological filtering or conditional refinements are performed.
4. Evaluation: The predicted segmentation mask is compared to the ground truth using the above standard evaluation metrics.

## 4. Results and Discussion

### 4.1. Implementation Details

The experiments were run in Google Colab environments with the A100, L4 GPU, and on a personal computer with GTX 1650 GPU using Python version 3.10.13, PyTorch version 2.1.2, and TorchVision version 0.16.2.

All models were trained using a dataset for each medical image database. The dataset was randomly partitioned into three subsets for training, validation, and testing, divided into 60%, 10%, and 30%. For the database of 134 images, the subsets comprise 80 images for training, 13 for validation, and 41 for testing. For the other database of 616 images, the subsets comprise 369 images for training, 61 for validation, and 186 for testing. In addition, data augmentation is applied to the training subsets of each database using vertical and horizontal mirror techniques and rotation at different degrees, increasing the number of images from 80 to 560 and from 369 to 2583.

For training, a batch size of four was selected and optimized for 150 epochs, with a learning rate of 0.01. The optimizer is AdamW, with a weight decay of 0.01 and the binary cross-entropy (BCE) loss function.

### 4.2. Quantitative Results

After training, the best model for each dataset (concerning the best validation loss) was tested to measure its performance in the five evaluation metrics. Moreover, each model was visually inspected.

Table 1 presents the segmentation performance of different U-Net variants on the DCA1 dataset. Among the evaluated models, U-Net-16 consistently achieves the best overall performance, obtaining the highest values for IoU (0.55676), dice (0.70972), accuracy (0.96964), recall (0.67613), and F1-score (0.71855). Compared to the Vanilla U-Net, U-Net-16 improves IoU by 15.96% (from 0.48023 to 0.55676) and Dice by 12.58% (from 0.63043 to 0.70972), indicating a more precise segmentation. Similarly, Recall increases by 18.75% (from 0.56936 to 0.67613), and the F1-score improves by 9.87% (from 0.65387 to 0.71855), highlighting a better balance between false positives and false negatives. However, precision (0.77341) is slightly lower than that of the Vanilla U-Net (0.79636), suggesting that while U-Net-16 is more effective in detecting vessels, it introduces a minor increase in false positives.

Similarly, Table 2 presents the results for the ICA dataset, where U-Net-16 is also the best-performing model, achieving the highest values for IoU (0.77315), dice (0.87010), accuracy (0.98554), recall (0.86267), and F1-score (0.87470). Compared to Vanilla U-Net, U-Net-16 improves IoU by 1.46% (from 0.76198 to 0.77315), and dice by 0.85% (from 0.86277 to 0.87010). Additionally, recall increases by 1.80% (from 0.84741 to 0.86267), and the F1-score improves by 0.80% (from 0.86773 to 0.87470), demonstrating consistent improvements in segmentation quality.

Notably, U-Net-32 exhibits the highest precision (0.89698) in the ICA dataset, but with a slightly lower recall (0.84778), suggesting a trade-off between false positives and false negatives. While U-Net-32 is more conservative in its segmentations, U-Net-16 provides a better balance between precision and recall, making it the most robust model for vessel segmentation across both datasets.

These results confirm that U-Net-16 is the best-performing model in both datasets. Demonstrating that pruning U-Net architectures enhances segmentation metrics while maintaining a compact and efficient model structure.

### 4.3. Visual Results

**Figure 4:**
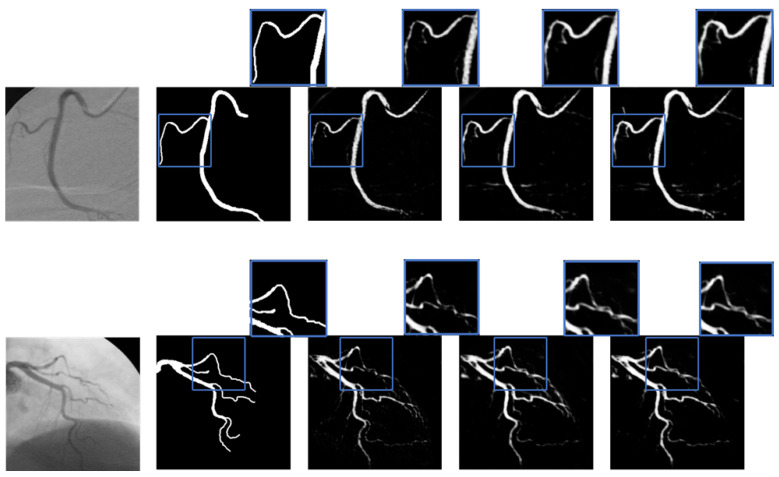
Prediction images of different U-Net configurations on the DCA1 dataset. From left to right: original XCA image, ground truth, vanilla U-Net, U-Net-32, and U-Net-16. The blue squares mark an obvious difference in the segmentation.

Visually, Figure 4 presents the segmentation masks of the DCA1 dataset compared to the predictions generated by the different models. Notably, as the number of filters is reduced, the models demonstrate improved generalization, particularly in the segmentation of thin blood vessel structures and bifurcations (highlighted in blue squares in the figure). This suggests that pruning enhances the model’s ability to capture fine details, which are critical for accurate vascular segmentation.

**Figure 5:**
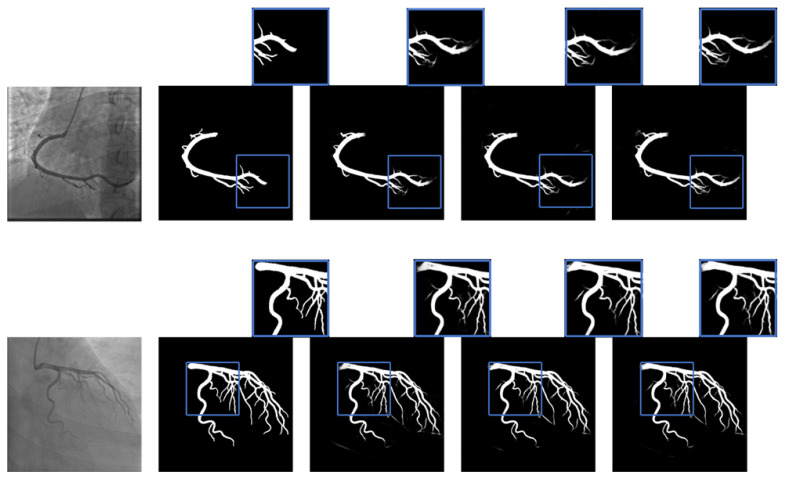
Prediction images of different U-Net configurations on the ICA dataset. From left to right: original XCA image, ground truth, vanilla U-Net, U-Net-32, and U-Net-16. The blue squares mark an obvious difference in the segmentation.

Similarly, Figure 5 illustrates the segmentation masks of the ICA dataset and their corresponding predictions. The pruned models, especially U-Net-16, not only improves segmentation in thin vessels and bifurcations but also enhances the delineation of thicker vessels, ensuring more complete vessel filling. This improvement indicates that kernel pruning helps the model better differentiate vessel structures across various thicknesses, leading to a more anatomically accurate segmentation.

Despite being a more complex model, the Vanilla U-Net exhibits lower performance metrics compared to the pruned versions. This outcome can be attributed to its increased number of parameters combined with the limited size of the training dataset, which may cause the model to focus on general image characteristics rather than fine vessel structures. As a result, its ability to generalize well to thin vessels, junctions, and bifurcations is reduced, reinforcing the effectiveness of the pruning strategy.

### 4.4. Discussion

Both numerical and visual results demonstrate that the employed pruning approach improves segmentation accuracy while significantly reducing the model size. Notably, U-Net-16 achieves high segmentation performance while requiring only 1.9 M parameters, making it a highly efficient alternative for lightweight deployment in resource-constrained environments.

However, one limitation of this approach is the uniform reduction in filters across all layers, which may not be the most optimal pruning strategy. A more adaptive approach, selectively pruning different layers based on their contribution to segmentation accuracy, could potentially yield a smaller yet equally or more effective subnetwork than U-Net-16.

Despite this limitation, this work establishes a strong foundation for developing lightweight models for XCA segmentation, demonstrating that kernel pruning can lead to models that are not only computationally efficient but also capable of achieving high segmentation accuracy. Future research may explore adaptive pruning techniques or hybrid strategies, such as integrating attention mechanisms, to further enhance segmentation quality while maintaining efficiency.

## 5. Conclusions and Outlook

This study introduces a kernel pruning strategy for constructing lightweight U-Net architectures tailored to coronary vessel segmentation in XCA images. By systematically reducing the number of filters in the vanilla U-Net model, it demonstrates that segmentation accuracy can be preserved—or even enhanced—while significantly decreasing the model’s complexity. Notably, the pruned model (U-Net-16) maintains only 1.9M parameters, compared to the original model’s 31M, offering a 94% reduction in model size. Empirical results across two benchmark datasets, DCA1 and ICA, validate the effectiveness of the proposed approach. On the DCA1 dataset, the pruned U-Net-16 achieved the highest performance, with an IoU of 0.5568 (↑15.96%), dice score of 0.7097 (↑12.58%), recall of 0.6761 (↑18.75%), and F1-score of 0.7186 (↑9.87%) compared to the vanilla U-Net. These improvements demonstrate the model’s enhanced ability to capture thin vessels, bifurcations, and fine vascular structures. On the ICA dataset, U-Net-16 also attained the best results, with an IoU of 0.7732 (↑1.46%), dice score of 0.8701 (↑0.85%), recall of 0.8627 (↑1.80%), and F1-score of 0.8747 (↑0.80%) over the vanilla U-Net. This work contributes new insights into the role of structured kernel pruning as a viable strategy for optimizing medical image segmentation networks, contrasting with the prevailing trend of increasing model complexity.

However, a current limitation is the uniform pruning across all layers, which may not optimally preserve discriminative features at different levels of abstraction. Future work will explore adaptive pruning strategies that dynamically adjust filter reduction based on the importance of feature maps. Additionally, integrating attention mechanisms and multi-scale fusion could enhance segmentation accuracy, particularly in anatomically complex regions.

Beyond coronary angiography, this pruning framework can be extended to other medical imaging modalities, such as retinal vessel segmentation, brain tumor delineation, or lung nodule detection. Such extensions will help establish the generalization and clinical relevance of lightweight, pruned architectures for medical applications. 

## Figures and Tables

**Table 1:** Segmentation result for the DCA1 dataset for different reduction α values.

Model	α	IoU	DICE	ACC	PREC	REC	F1
Vanilla U-Net	N/A	0.48023	0.63043	0.96612	**0.79636**	0.56936	0.65387
U-Net-32	2	0.53645	0.69195	0.96824	0.77203	0.65185	0.70216
U-Net-16	4	**0.55676**	**0.70972**	**0.96964**	0.77341	**0.67613**	**0.71855**

The best metrics are highlighted in bold.

**Table 2:** Segmentation result for the ICA dataset for different reduction α values.

Model	α	IoU	DICE	ACC	PREC	REC	F1
Vanilla U-Net	N/A	0.76198	0.86277	0.98489	0.89085	0.84741	0.86773
U-Net-32	2	0.76645	0.86572	0.98526	**0.89698**	0.84778	0.87082
U-Net-16	4	**0.77315**	**0.87010**	**0.98554**	0.88879	**0.86267**	**0.87470**

The best metrics are highlighted in bold.

## Data Availability

The datasets are available in their original repository. For DCA1: http://personal.cimat.mx:8181/~ivan.cruz/Databases/DB_Angiograms_134.zip, accessed on 27 March 2025. and for ICA: https://github.com/MIILab-MTU/ICA_NJ_BinarySeg, accessed on 27 March 2025.

## Abbreviations

The following abbreviations are used in this manuscript:

APN	Angiographic Processing Network
BCE	Binary Cross-Entropy
CAD	Coronary Artery Disease
CNN	Convolutional Neural Network
LCA	Left Coronary Artery
ReLU	Rectified Linear Unit
RCA	Right Coronary Artery
XCA	X-ray Coronary Angiography
